# Aqua­bis­(3-fluoro­benzoato-κ*O*)(1,10-phenanthroline-κ^2^ *N*,*N*′)copper(II)

**DOI:** 10.1107/S1600536811012220

**Published:** 2011-04-13

**Authors:** Xin Yin

**Affiliations:** a Department of Chemistry, East China University of Science and Technology, School of Chemistry and Molecular Engineering, Mei Long Road 130, Shanghai 200237, People’s Republic of China

## Abstract

In the title compound, [Cu(C_7_H_4_FO_2_)_2_(C_12_H_8_N_2_)(H_2_O)], the coordination around the Cu^II^ atom is square-pyramidal. The equatorial positions are occupied by two N atoms from a 1,10-phenanthroline ligand [Cu—N = 2.008 (3) and 2.019 (3) Å] and two O atoms from 3-fluoro­benzoate ligands and a water mol­ecule [Cu—O = 1.950 (2) and 1.978 (2) Å]. One O atom from another 3-fluoro­benzoate ligand occupies the apical positon [Cu—O = 2.210 (2) Å]. Hydrogen bonds occur between coordinated water mol­ecules and benzoate ligands, while O—H⋯O, C—H⋯O, C—H⋯F and π–π stacking [centroid–centroid distance = 3.731 (2) Å] inter­actions consolidate the crystal packing.

## Related literature

A number of copper SOD mimetics (SOD = superoxide dismutase) have been shown to possess anti­tumor activity and have been proposed as a new class of potential anti­cancer agents, see: Devereux *et al.* (2007 ▶). Phen­oxy­alkanoic acids inter­act with Cu(II) ions to form complexes which have been shown to have diverse stereochemistries, see: Smith *et al.* (1981 ▶, 1982 ▶). For the structures of similar coordination compounds, see: Liu *et al.* (2009 ▶); Zhu & Xiao (2006 ▶).
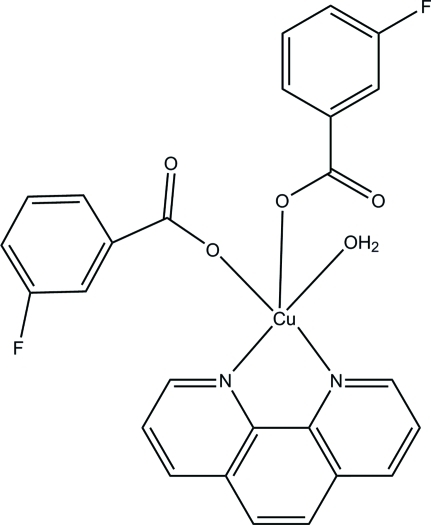


## Experimental

### 

#### Crystal data

[Cu(C_7_H_4_FO_2_)_2_(C_12_H_8_N_2_)(H_2_O)]
*M* *_r_* = 539.96
Triclinic, 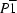

*a* = 9.9914 (8) Å
*b* = 10.7258 (9) Å
*c* = 11.6166 (10) Å
α = 73.208 (1)°
β = 70.082 (1)°
γ = 86.293 (1)°
*V* = 1119.74 (16) Å^3^
*Z* = 2
Mo *K*α radiation
μ = 1.04 mm^−1^
*T* = 295 K
0.25 × 0.20 × 0.15 mm

#### Data collection

Bruker APEXII CCD diffractometer
Absorption correction: multi-scan (*SADABS*; Sheldrick, 2003 ▶) *T*_min_ = 0.782, *T*_max_ = 0.860
5876 measured reflections
3972 independent reflections
2924 reflections with *I* > 2σ(*I*)
*R*_int_ = 0.058

#### Refinement

*R*[*F* ^2^ > 2σ(*F* ^2^)] = 0.046
*wR*(*F* ^2^) = 0.132
*S* = 1.03
3972 reflections
325 parameters
H-atom parameters constrained
Δρ_max_ = 0.99 e Å^−3^
Δρ_min_ = −0.47 e Å^−3^

### 

Data collection: *SMART* (Bruker, 2001 ▶); cell refinement: *SAINT-Plus* (Bruker, 2003 ▶); data reduction: *SAINT-Plus*; program(s) used to solve structure: *SHELXTL* (Sheldrick, 2008 ▶); program(s) used to refine structure: *SHELXTL*; molecular graphics: *SHELXTL*; software used to prepare material for publication: *SHELXTL*.

## Supplementary Material

Crystal structure: contains datablocks I, global. DOI: 10.1107/S1600536811012220/hg5016sup1.cif

Structure factors: contains datablocks I. DOI: 10.1107/S1600536811012220/hg5016Isup2.hkl

Additional supplementary materials:  crystallographic information; 3D view; checkCIF report

## Figures and Tables

**Table 1:** Hydrogen-bond geometry (Å, °)

*D*—H⋯*A*	*D*—H	H⋯*A*	*D*⋯*A*	*D*—H⋯*A*
O1*W*—H1*W*⋯O1	0.85	1.75	2.585 (4)	163
O1*W*—H2*W*⋯O4	0.85	1.80	2.612 (4)	161
C10—H10⋯O3	0.93	2.53	3.005 (4)	112
C1—H1⋯F1^i^	0.93	2.33	3.213 (6)	158
C8—H8⋯O4^ii^	0.93	2.39	3.309 (4)	171

Symmetry codes: (i) 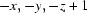; (ii) 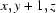.

**Fig. 1.**
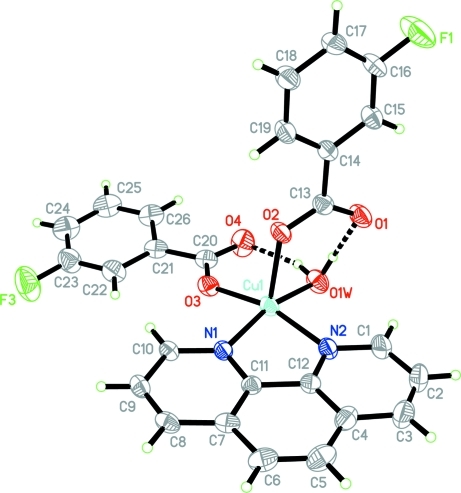
The molecular structure of (I), (thermal ellipsoids are shown at 30% probability levels).

## supplementary crystallographic information

### Comment

A number of copper SOD mimetics have been shown to possess antitumor activity and have been proposed as a new class of potential anticancer agents (Devereux *et al.* 2007). Morever, phenoxyalkanoic acids interact with Cu(II) ions to form complexes which have been shown to have diverse stereochemistries (Smith *et al.* 1981,1982). Studying structure of such copper complexes is important to the understanding of copper biochemistry. Therefore, we have synthesized the title compound, (I), and report its crystal structure here.

In the title monomer complex, the copper atom adopts a square pyramidal environment defined by two nitrogen donors from the 1,10-phenanthroline ligand, two carboxyl oxygen atoms from two 3-fluorobenzoate ligands and one oxygen atom from the coordinated water molecule O atom (Fig. 1). Atoms N1, N2, O3, and O1w are sitting on the basal plane, while atom O2 occupies the apical position. Each 3-fluorobenzoate ligand is mono-coordinated to the metal atom. The coordinated water molecule acts as double donor to the carbonyl groups of the 3-fluorobenzoate ligands, forming two intramolecular O-H···O hydrogen bonds (Table 1), which consolidates the solid structure. The crystal packing exhibits also weak intermolecularC—H···O hydrogen bonds, π–π interactions and short intermolecular C—H···F Contacts. Similar coordination is observed in other Cu structures (Liu *et al.*,2009; Zhu *et al.*, 2006).

### Experimental

All reagents were obtained from commercial sources and used without further purification. CuCl_2_.2H_2_O (0.017 g, 0.10 mmol) was successively added to 20 ml CH_3_OH, H_2_O (1:1, *v*/*v*)solution. Then 3-fluorobenzoic acid (0.028 g, 0.20 mmol) and 1,10-phenanthroline (0.017 g, 0.10 mmol) were subsequently added. The pH value of the mixture was adjusted to about 5.5 with NaOH solution and stirred continuously for 1 h to give a blue clear solution. After filtration, the blue filtrate was allowed to stand at room temperature for one week to give blue block-shaped crystals suitable for X-ray analysis.Analysis required for C_26_H_18_CuF_2_N_2_O_5_: *C* 57.83, H 3.36, N 5.19%; found: C 57.64, H 3.58, N 5.09%. m.p. 463.5-464 K.

### Refinement

All C-bound H atoms were positioned geometrically and treated as riding, with C—H = 0.93Å and *U*_iso_(H) = 1.2Ueq(C). The water H atoms were found in a difference Fourier map and refined freely.

### Crystal data

**Table:**

[Cu(C_7_H_4_FO_2_)_2_(C_12_H_8_N_2_)(H_2_O)]	*Z* = 2
*M**_r_* = 539.96	*F*(000) = 550
Triclinic, *P*1	*D*_x_ = 1.602 Mg m^−^^3^
Hall symbol: -P 1	Mo *K*α radiation, λ = 0.71073 Å
*a* = 9.9914 (8) Å	Cell parameters from 2269 reflections
*b* = 10.7258 (9) Å	θ = 2.4–23.9°
*c* = 11.6166 (10) Å	µ = 1.04 mm^−^^1^
α = 73.208 (1)°	*T* = 295 K
β = 70.082 (1)°	Block, blue
γ = 86.293 (1)°	0.25 × 0.20 × 0.15 mm
*V* = 1119.74 (16) Å^3^	

### Data collection

**Table:**

Bruker APEXII CCD diffractometer	3972 independent reflections
Radiation source: fine-focus sealed tube	2924 reflections with *I* > 2σ(*I*)
graphite	*R*_int_ = 0.058
φ and ω scans	θ_max_ = 25.2°, θ_min_ = 1.9°
Absorption correction: multi-scan (*SADABS*; Sheldrick, 2003)	*h* = −11→10
*T*_min_ = 0.782, *T*_max_ = 0.860	*k* = −12→12
5876 measured reflections	*l* = −13→9

### Refinement

**Table:**

Refinement on *F*^2^	Primary atom site location: structure-invariant direct methods
Least-squares matrix: full	Secondary atom site location: difference Fourier map
*R*[*F*^2^ > 2σ(*F*^2^)] = 0.046	Hydrogen site location: inferred from neighbouring sites
*wR*(*F*^2^) = 0.132	H-atom parameters constrained
*S* = 1.03	*w* = 1/[σ^2^(*F*_o_^2^) + (0.0675*P*)^2^] where *P* = (*F*_o_^2^ + 2*F*_c_^2^)/3
3972 reflections	(Δ/σ)_max_ < 0.001
325 parameters	Δρ_max_ = 0.99 e Å^−^^3^
0 restraints	Δρ_min_ = −0.47 e Å^−^^3^

### Special details

**Table:**

Geometry. All e.s.d.'s (except the e.s.d. in the dihedral angle between two l.s. planes) are estimated using the full covariance matrix. The cell e.s.d.'s are taken into account individually in the estimation of e.s.d.'s in distances, angles and torsion angles; correlations between e.s.d.'s in cell parameters are only used when they are defined by crystal symmetry. An approximate (isotropic) treatment of cell e.s.d.'s is used for estimating e.s.d.'s involving l.s. planes.
Refinement. Refinement of *F*^2^ against ALL reflections. The weighted *R*-factor *wR* and goodness of fit *S* are based on *F*^2^, conventional *R*-factors *R* are based on *F*, with *F* set to zero for negative *F*^2^. The threshold expression of *F*^2^ > σ(*F*^2^) is used only for calculating *R*-factors(gt) *etc*. and is not relevant to the choice of reflections for refinement. *R*-factors based on *F*^2^ are statistically about twice as large as those based on *F*, and *R*- factors based on ALL data will be even larger.

### Fractional atomic coordinates and isotropic or equivalent isotropic displacement parameters (Å^2^)

**Table:**

	*x*	*y*	*z*	*U*_iso_*/*U*_eq_	
C1	−0.1039 (5)	0.3825 (4)	0.2313 (4)	0.0699 (13)	
H1	−0.1100	0.2939	0.2400	0.084*	
C2	−0.2204 (5)	0.4403 (6)	0.2961 (5)	0.0823 (15)	
H2	−0.3030	0.3900	0.3478	0.099*	
C3	−0.2174 (5)	0.5693 (5)	0.2861 (4)	0.0766 (14)	
H3	−0.2971	0.6075	0.3301	0.092*	
C4	−0.0929 (4)	0.6439 (4)	0.2089 (4)	0.0575 (10)	
C5	−0.0752 (5)	0.7814 (5)	0.1881 (4)	0.0686 (12)	
H5	−0.1507	0.8262	0.2288	0.082*	
C6	0.0486 (5)	0.8471 (4)	0.1106 (4)	0.0610 (11)	
H6	0.0569	0.9360	0.0995	0.073*	
C7	0.1661 (4)	0.7829 (3)	0.0457 (4)	0.0460 (9)	
C8	0.2980 (4)	0.8451 (3)	−0.0358 (4)	0.0503 (9)	
H8	0.3121	0.9341	−0.0512	0.060*	
C9	0.4045 (4)	0.7747 (3)	−0.0918 (3)	0.0487 (9)	
H9	0.4912	0.8159	−0.1471	0.058*	
C10	0.3850 (4)	0.6403 (3)	−0.0670 (3)	0.0423 (8)	
H10	0.4601	0.5931	−0.1045	0.051*	
C11	0.1545 (4)	0.6493 (3)	0.0640 (3)	0.0400 (8)	
C12	0.0226 (4)	0.5790 (4)	0.1455 (3)	0.0456 (9)	
C13	0.2143 (4)	0.2156 (3)	0.3195 (3)	0.0470 (9)	
C14	0.2732 (4)	0.1690 (3)	0.4278 (3)	0.0432 (8)	
C15	0.2137 (4)	0.0593 (4)	0.5261 (3)	0.0516 (9)	
H15	0.1346	0.0157	0.5289	0.062*	
C16	0.2740 (5)	0.0158 (4)	0.6198 (4)	0.0584 (11)	
C17	0.3895 (4)	0.0746 (4)	0.6215 (4)	0.0569 (10)	
H17	0.4276	0.0405	0.6864	0.068*	
C18	0.4494 (5)	0.1861 (4)	0.5246 (4)	0.0603 (11)	
H18	0.5270	0.2299	0.5241	0.072*	
C19	0.3909 (4)	0.2315 (4)	0.4279 (4)	0.0538 (10)	
H19	0.4316	0.3056	0.3615	0.065*	
C20	0.4248 (4)	0.2594 (3)	−0.1064 (4)	0.0503 (9)	
C21	0.5686 (4)	0.2694 (4)	−0.2072 (4)	0.0514 (10)	
C22	0.6404 (5)	0.3875 (4)	−0.2736 (4)	0.0632 (11)	
H22	0.6000	0.4637	−0.2567	0.076*	
C23	0.7713 (5)	0.3922 (5)	−0.3647 (4)	0.0757 (14)	
C24	0.8378 (6)	0.2835 (5)	−0.3942 (5)	0.0802 (14)	
H24	0.9261	0.2901	−0.4580	0.096*	
C25	0.7682 (6)	0.1643 (5)	−0.3252 (5)	0.0765 (14)	
H25	0.8108	0.0883	−0.3406	0.092*	
C26	0.6352 (5)	0.1575 (4)	−0.2331 (4)	0.0640 (12)	
H26	0.5893	0.0765	−0.1876	0.077*	
Cu1	0.20778 (5)	0.38751 (4)	0.05953 (4)	0.04433 (19)	
F1	0.2173 (4)	−0.0908 (3)	0.7161 (3)	0.1306 (14)	
F3	0.8389 (4)	0.5091 (3)	−0.4302 (3)	0.1242 (13)	
N1	0.2605 (3)	0.5780 (3)	0.0096 (3)	0.0395 (7)	
N2	0.0190 (3)	0.4496 (3)	0.1555 (3)	0.0498 (8)	
O1	0.1097 (3)	0.1514 (3)	0.3257 (3)	0.0654 (8)	
O2	0.2767 (3)	0.3139 (2)	0.2280 (2)	0.0486 (6)	
O3	0.3809 (3)	0.3625 (2)	−0.0732 (2)	0.0494 (6)	
O4	0.3565 (3)	0.1534 (3)	−0.0605 (3)	0.0765 (9)	
O1W	0.1185 (3)	0.2161 (2)	0.0909 (3)	0.0591 (7)	
H1W	0.1159	0.1795	0.1675	0.089*	
H2W	0.1895	0.1784	0.0524	0.089*	

### Atomic displacement parameters (Å^2^)

**Table:**

	*U*^11^	*U*^22^	*U*^33^	*U*^12^	*U*^13^	*U*^23^
C1	0.053 (3)	0.068 (3)	0.073 (3)	−0.016 (2)	−0.019 (2)	0.006 (2)
C2	0.044 (3)	0.104 (4)	0.071 (3)	−0.013 (3)	−0.004 (2)	0.001 (3)
C3	0.048 (3)	0.104 (4)	0.062 (3)	0.004 (3)	−0.005 (2)	−0.015 (3)
C4	0.045 (2)	0.076 (3)	0.052 (2)	0.007 (2)	−0.017 (2)	−0.020 (2)
C5	0.065 (3)	0.079 (3)	0.077 (3)	0.022 (2)	−0.028 (3)	−0.043 (3)
C6	0.070 (3)	0.055 (2)	0.074 (3)	0.018 (2)	−0.035 (2)	−0.033 (2)
C7	0.059 (2)	0.0390 (19)	0.050 (2)	0.0075 (17)	−0.0283 (19)	−0.0163 (17)
C8	0.069 (3)	0.0305 (18)	0.055 (2)	−0.0027 (18)	−0.028 (2)	−0.0089 (17)
C9	0.054 (2)	0.0401 (19)	0.047 (2)	−0.0096 (17)	−0.0141 (18)	−0.0052 (17)
C10	0.048 (2)	0.0339 (18)	0.042 (2)	−0.0013 (15)	−0.0135 (17)	−0.0069 (15)
C11	0.042 (2)	0.0411 (19)	0.0388 (19)	0.0018 (15)	−0.0186 (16)	−0.0084 (16)
C12	0.043 (2)	0.051 (2)	0.041 (2)	0.0009 (17)	−0.0171 (17)	−0.0057 (17)
C13	0.052 (2)	0.040 (2)	0.046 (2)	0.0026 (17)	−0.0149 (18)	−0.0108 (18)
C14	0.050 (2)	0.0377 (18)	0.0373 (19)	0.0029 (16)	−0.0101 (16)	−0.0094 (15)
C15	0.062 (3)	0.047 (2)	0.044 (2)	−0.0095 (18)	−0.0163 (19)	−0.0093 (18)
C16	0.080 (3)	0.045 (2)	0.040 (2)	−0.009 (2)	−0.020 (2)	0.0035 (18)
C17	0.068 (3)	0.057 (2)	0.046 (2)	0.001 (2)	−0.025 (2)	−0.0077 (19)
C18	0.067 (3)	0.059 (2)	0.049 (2)	−0.002 (2)	−0.020 (2)	−0.007 (2)
C19	0.059 (2)	0.047 (2)	0.046 (2)	−0.0093 (18)	−0.0140 (19)	−0.0020 (18)
C20	0.062 (3)	0.042 (2)	0.057 (2)	0.0039 (18)	−0.032 (2)	−0.0160 (19)
C21	0.068 (3)	0.044 (2)	0.055 (2)	0.0104 (19)	−0.030 (2)	−0.0217 (19)
C22	0.077 (3)	0.055 (2)	0.054 (3)	0.012 (2)	−0.015 (2)	−0.021 (2)
C23	0.080 (3)	0.069 (3)	0.062 (3)	0.005 (3)	−0.007 (3)	−0.016 (3)
C24	0.084 (4)	0.096 (4)	0.066 (3)	0.024 (3)	−0.024 (3)	−0.038 (3)
C25	0.092 (4)	0.075 (3)	0.085 (4)	0.027 (3)	−0.041 (3)	−0.049 (3)
C26	0.079 (3)	0.055 (2)	0.077 (3)	0.011 (2)	−0.039 (3)	−0.034 (2)
Cu1	0.0507 (3)	0.0328 (3)	0.0478 (3)	−0.00640 (18)	−0.0178 (2)	−0.00571 (19)
F1	0.169 (3)	0.110 (2)	0.103 (2)	−0.072 (2)	−0.081 (2)	0.043 (2)
F3	0.113 (3)	0.091 (2)	0.110 (3)	−0.0060 (19)	0.029 (2)	−0.018 (2)
N1	0.0405 (16)	0.0362 (15)	0.0383 (16)	−0.0007 (13)	−0.0112 (13)	−0.0076 (13)
N2	0.0462 (19)	0.0481 (18)	0.0471 (18)	−0.0098 (14)	−0.0152 (15)	0.0005 (14)
O1	0.0704 (19)	0.0583 (16)	0.0621 (18)	−0.0245 (14)	−0.0326 (16)	0.0093 (14)
O2	0.0559 (16)	0.0416 (13)	0.0425 (14)	−0.0088 (11)	−0.0175 (12)	0.0004 (12)
O3	0.0595 (16)	0.0316 (12)	0.0563 (16)	0.0019 (11)	−0.0172 (13)	−0.0142 (12)
O4	0.076 (2)	0.0439 (15)	0.110 (3)	−0.0078 (15)	−0.0196 (18)	−0.0350 (17)
O1W	0.0714 (19)	0.0472 (14)	0.0597 (17)	−0.0153 (13)	−0.0244 (15)	−0.0100 (13)

### Geometric parameters (Å, °)

**Table:**

C1—N2	1.347 (5)	C15—H15	0.9300
C1—C2	1.374 (6)	C16—F1	1.338 (4)
C1—H1	0.9300	C16—C17	1.359 (6)
C2—C3	1.357 (7)	C17—C18	1.383 (5)
C2—H2	0.9300	C17—H17	0.9300
C3—C4	1.395 (6)	C18—C19	1.391 (5)
C3—H3	0.9300	C18—H18	0.9300
C4—C12	1.404 (5)	C19—H19	0.9300
C4—C5	1.437 (6)	C20—O4	1.243 (4)
C5—C6	1.350 (6)	C20—O3	1.277 (4)
C5—H5	0.9300	C20—C21	1.500 (5)
C6—C7	1.419 (5)	C21—C22	1.375 (5)
C6—H6	0.9300	C21—C26	1.394 (5)
C7—C11	1.393 (5)	C22—C23	1.367 (6)
C7—C8	1.407 (5)	C22—H22	0.9300
C8—C9	1.354 (5)	C23—F3	1.347 (5)
C8—H8	0.9300	C23—C24	1.376 (6)
C9—C10	1.398 (5)	C24—C25	1.379 (7)
C9—H9	0.9300	C24—H24	0.9300
C10—N1	1.340 (4)	C25—C26	1.384 (6)
C10—H10	0.9300	C25—H25	0.9300
C11—N1	1.351 (4)	C26—H26	0.9300
C11—C12	1.438 (5)	Cu1—O3	1.950 (2)
C12—N2	1.360 (5)	Cu1—O1W	1.978 (2)
C13—O1	1.259 (4)	Cu1—N1	2.008 (3)
C13—O2	1.272 (4)	Cu1—N2	2.019 (3)
C13—C14	1.510 (5)	Cu1—O2	2.210 (2)
C14—C15	1.376 (5)	O1W—H1W	0.8545
C14—C19	1.392 (5)	O1W—H2W	0.8462
C15—C16	1.373 (5)		
			
N2—C1—C2	122.3 (4)	C18—C17—H17	120.7
N2—C1—H1	118.9	C17—C18—C19	118.6 (4)
C2—C1—H1	118.9	C17—C18—H18	120.7
C3—C2—C1	121.2 (4)	C19—C18—H18	120.7
C3—C2—H2	119.4	C18—C19—C14	121.7 (3)
C1—C2—H2	119.4	C18—C19—H19	119.1
C2—C3—C4	119.0 (4)	C14—C19—H19	119.1
C2—C3—H3	120.5	O4—C20—O3	124.3 (4)
C4—C3—H3	120.5	O4—C20—C21	118.9 (3)
C3—C4—C12	117.0 (4)	O3—C20—C21	116.8 (3)
C3—C4—C5	125.0 (4)	C22—C21—C26	118.1 (4)
C12—C4—C5	118.0 (4)	C22—C21—C20	121.5 (3)
C6—C5—C4	121.5 (4)	C26—C21—C20	120.4 (4)
C6—C5—H5	119.3	C23—C22—C21	119.6 (4)
C4—C5—H5	119.3	C23—C22—H22	120.2
C5—C6—C7	121.2 (4)	C21—C22—H22	120.2
C5—C6—H6	119.4	F3—C23—C22	118.7 (4)
C7—C6—H6	119.4	F3—C23—C24	118.0 (4)
C11—C7—C8	116.4 (3)	C22—C23—C24	123.4 (5)
C11—C7—C6	119.4 (4)	C23—C24—C25	117.3 (5)
C8—C7—C6	124.2 (4)	C23—C24—H24	121.3
C9—C8—C7	119.7 (3)	C25—C24—H24	121.3
C9—C8—H8	120.2	C24—C25—C26	120.2 (4)
C7—C8—H8	120.2	C24—C25—H25	119.9
C8—C9—C10	120.4 (3)	C26—C25—H25	119.9
C8—C9—H9	119.8	C25—C26—C21	121.4 (4)
C10—C9—H9	119.8	C25—C26—H26	119.3
N1—C10—C9	121.5 (3)	C21—C26—H26	119.3
N1—C10—H10	119.2	O3—Cu1—O1W	94.85 (10)
C9—C10—H10	119.2	O3—Cu1—N1	90.11 (10)
N1—C11—C7	124.4 (3)	O1W—Cu1—N1	165.91 (11)
N1—C11—C12	115.9 (3)	O3—Cu1—N2	164.15 (11)
C7—C11—C12	119.7 (3)	O1W—Cu1—N2	90.67 (12)
N2—C12—C4	123.9 (3)	N1—Cu1—N2	81.29 (11)
N2—C12—C11	115.9 (3)	O3—Cu1—O2	99.65 (10)
C4—C12—C11	120.2 (4)	O1W—Cu1—O2	91.54 (10)
O1—C13—O2	125.3 (3)	N1—Cu1—O2	100.63 (10)
O1—C13—C14	117.2 (3)	N2—Cu1—O2	95.04 (11)
O2—C13—C14	117.5 (3)	C10—N1—C11	117.6 (3)
C15—C14—C19	118.9 (4)	C10—N1—Cu1	128.7 (2)
C15—C14—C13	119.7 (3)	C11—N1—Cu1	113.7 (2)
C19—C14—C13	121.4 (3)	C1—N2—C12	116.6 (4)
C16—C15—C14	118.3 (4)	C1—N2—Cu1	130.3 (3)
C16—C15—H15	120.8	C12—N2—Cu1	113.0 (2)
C14—C15—H15	120.8	C13—O2—Cu1	121.4 (2)
F1—C16—C17	117.1 (4)	C20—O3—Cu1	129.5 (2)
F1—C16—C15	119.0 (4)	Cu1—O1W—H1W	100.5
C17—C16—C15	123.9 (4)	Cu1—O1W—H2W	100.5
C16—C17—C18	118.6 (4)	H1W—O1W—H2W	98.7
C16—C17—H17	120.7		
			
N2—C1—C2—C3	0.4 (8)	F3—C23—C24—C25	179.2 (5)
C1—C2—C3—C4	−0.3 (8)	C22—C23—C24—C25	−1.4 (8)
C2—C3—C4—C12	0.2 (7)	C23—C24—C25—C26	1.8 (7)
C2—C3—C4—C5	179.1 (5)	C24—C25—C26—C21	−0.3 (7)
C3—C4—C5—C6	−179.3 (4)	C22—C21—C26—C25	−1.6 (6)
C12—C4—C5—C6	−0.4 (6)	C20—C21—C26—C25	−179.6 (4)
C4—C5—C6—C7	0.4 (7)	C9—C10—N1—C11	−1.0 (5)
C5—C6—C7—C11	−1.1 (6)	C9—C10—N1—Cu1	−179.8 (3)
C5—C6—C7—C8	−179.7 (4)	C7—C11—N1—C10	−0.1 (5)
C11—C7—C8—C9	0.3 (5)	C12—C11—N1—C10	179.9 (3)
C6—C7—C8—C9	179.0 (4)	C7—C11—N1—Cu1	178.9 (3)
C7—C8—C9—C10	−1.3 (6)	C12—C11—N1—Cu1	−1.1 (4)
C8—C9—C10—N1	1.7 (6)	O3—Cu1—N1—C10	−12.2 (3)
C8—C7—C11—N1	0.4 (5)	O1W—Cu1—N1—C10	−123.0 (5)
C6—C7—C11—N1	−178.4 (3)	N2—Cu1—N1—C10	−178.9 (3)
C8—C7—C11—C12	−179.6 (3)	O2—Cu1—N1—C10	87.6 (3)
C6—C7—C11—C12	1.7 (5)	O3—Cu1—N1—C11	168.9 (2)
C3—C4—C12—N2	0.0 (6)	O1W—Cu1—N1—C11	58.1 (5)
C5—C4—C12—N2	−179.0 (4)	N2—Cu1—N1—C11	2.3 (2)
C3—C4—C12—C11	179.9 (4)	O2—Cu1—N1—C11	−91.2 (2)
C5—C4—C12—C11	0.9 (6)	C2—C1—N2—C12	−0.2 (6)
N1—C11—C12—N2	−1.7 (5)	C2—C1—N2—Cu1	175.7 (3)
C7—C11—C12—N2	178.3 (3)	C4—C12—N2—C1	0.0 (5)
N1—C11—C12—C4	178.4 (3)	C11—C12—N2—C1	−179.9 (3)
C7—C11—C12—C4	−1.6 (5)	C4—C12—N2—Cu1	−176.5 (3)
O1—C13—C14—C15	1.1 (5)	C11—C12—N2—Cu1	3.5 (4)
O2—C13—C14—C15	−176.9 (3)	O3—Cu1—N2—C1	123.0 (4)
O1—C13—C14—C19	178.3 (4)	O1W—Cu1—N2—C1	12.5 (4)
O2—C13—C14—C19	0.3 (5)	N1—Cu1—N2—C1	−179.2 (4)
C19—C14—C15—C16	−0.4 (5)	O2—Cu1—N2—C1	−79.1 (4)
C13—C14—C15—C16	176.9 (3)	O3—Cu1—N2—C12	−61.0 (5)
C14—C15—C16—F1	−180.0 (4)	O1W—Cu1—N2—C12	−171.6 (3)
C14—C15—C16—C17	−0.1 (6)	N1—Cu1—N2—C12	−3.2 (2)
F1—C16—C17—C18	−179.0 (4)	O2—Cu1—N2—C12	96.8 (2)
C15—C16—C17—C18	1.1 (7)	O1—C13—O2—Cu1	−0.4 (5)
C16—C17—C18—C19	−1.6 (6)	C14—C13—O2—Cu1	177.4 (2)
C17—C18—C19—C14	1.1 (6)	O3—Cu1—O2—C13	−118.2 (3)
C15—C14—C19—C18	−0.1 (6)	O1W—Cu1—O2—C13	−23.0 (3)
C13—C14—C19—C18	−177.4 (3)	N1—Cu1—O2—C13	149.9 (3)
O4—C20—C21—C22	171.0 (4)	N2—Cu1—O2—C13	67.8 (3)
O3—C20—C21—C22	−9.2 (5)	O4—C20—O3—Cu1	3.8 (6)
O4—C20—C21—C26	−11.0 (6)	C21—C20—O3—Cu1	−176.0 (2)
O3—C20—C21—C26	168.8 (3)	O1W—Cu1—O3—C20	−10.8 (3)
C26—C21—C22—C23	1.9 (6)	N1—Cu1—O3—C20	−177.6 (3)
C20—C21—C22—C23	179.9 (4)	N2—Cu1—O3—C20	−120.8 (4)
C21—C22—C23—F3	179.0 (4)	O2—Cu1—O3—C20	81.6 (3)
C21—C22—C23—C24	−0.5 (8)		

### Hydrogen-bond geometry (Å, °)

**Table:**

*D*—H···*A*	*D*—H	H···*A*	*D*···*A*	*D*—H···*A*
O1W—H1W···O1	0.85	1.75	2.585 (4)	163
O1W—H2W···O4	0.85	1.80	2.612 (4)	161
C10—H10···O3	0.93	2.53	3.005 (4)	112
C1—H1···F1^i^	0.93	2.33	3.213 (6)	158.
C8—H8···O4^ii^	0.93	2.39	3.309 (4)	171.

Symmetry codes: (i) −*x*, −*y*, −*z*+1; (ii) *x*, *y*+1, *z*.
